# The bromodomain inhibitor N-methyl pyrrolidone reduced fat accumulation in an ovariectomized rat model

**DOI:** 10.1186/s13148-016-0209-2

**Published:** 2016-04-22

**Authors:** Bebeka Gjoksi, Chafik Ghayor, Indranil Bhattacharya, Marcy Zenobi-Wong, Franz E. Weber

**Affiliations:** Oral Biotechnology & Bioengineering, Center for Dental Medicine, Cranio-Maxillofacial and Oral Surgery, University of Zurich, Zurich, Switzerland; Department of Internal Medicine, University Hospital of Zurich, Zurich, Switzerland; Cartilage Engineering + Regeneration Laboratory, ETH Zurich, Zurich, Switzerland; CABMM, Center for Applied Biotechnology and Molecular Medicine, University of Zurich, Zurich, Switzerland; Zurich Center for Integrative Human Physiology (ZIHP), University of Zurich, Zurich, Switzerland

**Keywords:** Osteoporosis, Adipocytes, Epigenetic modulator, Small inhibitor molecule

## Abstract

**Background:**

Weight gain is one of the consequences of estrogen deficiency and constitutes a major health problem. The present study highlights the effects of N-methyl pyrrolidone (NMP) on adipogenesis in osteoporosis induced by estrogen deficiency in an ovariectomized rat model.

**Results:**

Ovariectomy resulted in body weight gain, increased femoral marrow adipocytes, and hypertrophic adipocytes in white adipose tissue, distorted serum leptin, and TNF-α and PPARγ levels. Treatment with NMP normalized these parameters similar to the control group. In vitro, NMP inhibited the differentiation of 3T3-L1 pre-adipocytes and hMSCs, indicating its anti-adipogenic effect. Moreover, PPARγ was significantly reduced with NMP treatment in in vivo and in vitro experiments. NMP inhibited BRD2 and BRD4 binding in an AlphaScreen assay, with an IC50 of 3 and 4 mM, respectively. The effect of NMP was consistent with its role as a bromodomain inhibitor.

**Conclusions:**

Our data indicates that NMP inhibits the adipogenic effect of estrogen deficiency at the level of PPARγ expression by BRD4 inhibition.

**Electronic supplementary material:**

The online version of this article (doi:10.1186/s13148-016-0209-2) contains supplementary material, which is available to authorized users.

## Background

Osteoporosis is the most common metabolic bone disease which is aggravated by estrogen deficiency. It is manifested as a systemic impairment of mass, strength, and micro-architecture of bone, which increases the predisposition to fractures as a result of bone fragility [1]. Additionally, estrogen deficiency is also associated with an increased risk of other metabolic diseases including obesity, heart disease, diabetes, and hypertension [2]. Estrogen levels in the body are very important since they regulate key features of metabolism such as food intake, body weight, glucose homeostasis/insulin, body fat distribution, lipolysis/lipogenesis, inflammation, reproduction, and cognition [3]. The increase in bodyweight/obesity in menopausal women accompanied with postmenopausal osteoporosis is a significant public health concern [4].

It is known that an inverse relationship exists between osteogenic and adipogenic programming. Multiple signaling pathways have been demonstrated to preferentially induce osteogenesis at the expense of adipogenesis, or vice versa. The ability of mesenchymal stem cells (MSCs) to differentiate in one or more of five mesenchymal lineages is determined by different factors such as hormones, cytokines, and growth factors [5]. There is a known but not a well-defined relationship between bone mass, bone strength, and bone marrow fat content. In normal state, the bone marrow balance of osteoblastic and adipocyte cell differentiation favors bone formation, while during osteoporosis, there is an increase in adipocyte formation [6]. Bone marrow adipose tissue is inversely associated with bone mineral density (BMD) in postmenopausal osteoporosis and other conditions such as old age and treatment with glucocorticoids medications [7, 8]. Data also suggests that estrogen plays an important role not only in the homeostasis of bone marrow fat but also in regulating body fat distribution in women. It has been shown that postmenopausal woman have higher total and abdominal fat mass [9] and lower lean body mass than premenopausal women [10]. Estrogen deficiency among menopaused elderly women leads to the development of visceral fat depots which are known to be associated with other severe diseases including diabetes mellitus type 2, the metabolic syndrome, and cardiovascular diseases, while bone is resorbed [11–14].

On the molecular level, peroxisome proliferator-activated receptors γ (PPARγ) is considered the master regulator of adipogenesis, and without it, the precursor cells are incapable of expressing any known aspect of adipocyte phenotype [15].

Epigenetic regulation is a known fundamental regulatory mechanism for normal development which causes heritable differences in cell behaviors [16]. Epigenetic therapy is of particular interest as a pluripotent approach to directly target different medical conditions. Bromo and extraterminal (BET)-protein bromodomain inhibitors are a novel epigenetic approach to treat a wide range of diseases. Bromodomains are protein motifs binding acetyl-lysine residues of nucleosomal histones or other proteins [17]. BET-proteins contain two bromodomain domains and an extraterminal domain [18]. They serve as scaffolding modules that recruit transcription regulatory factors to chromatin to form protein complexes which regulate gene transcription in response to signal transduction. BRD2 and BRD4, both members of the BET-protein family, were recently shown to play a critical role in adipogenesis [19–21].

In the last years, we showed that N-methyl pyrrolidone (NMP), a small molecule used as a drug solvent and as a constituent in FDA-approved medical devices, targets osteoblast and osteoclast differentiation [22, 23]. Indeed, NMP has demonstrated to have polyvalent capabilities; it enhances BMP-2-induced osteoblast differentiation and bone regeneration and disturbs osteoclast differentiation and bone resorption [22, 23]. Recently, in an animal model of osteoporosis, we showed that NMP exhibits an anti-osteoporotic capability [24] while in an in vitro inflammation model, NMP attenuated the production of LPS induced pro-inflammatory cytokines [25].

Osteoblasts and adipocytes differentiate from a common precursor, bone marrow mesenchymal stem cells (BMSCs) [26, 27]. There is an estrogen-linked reduction in osteogenesis that is accompanied by an increase in adipogenesis. This switch increases white adipocytes and decreases osteoblasts numbers, triggering estrogen deficiency-related bone loss [28]. The goal of this study was to determine the effect of NMP on adipogenesis in vitro and white fat tissue accumulation and marrow fat content in an in vivo model of estrogen deficiency-induced osteoporosis.

## Results

### Potency of NMP treatment on weight gain, bone marrow adiposity, and biomarkers

**Fig. 1:**
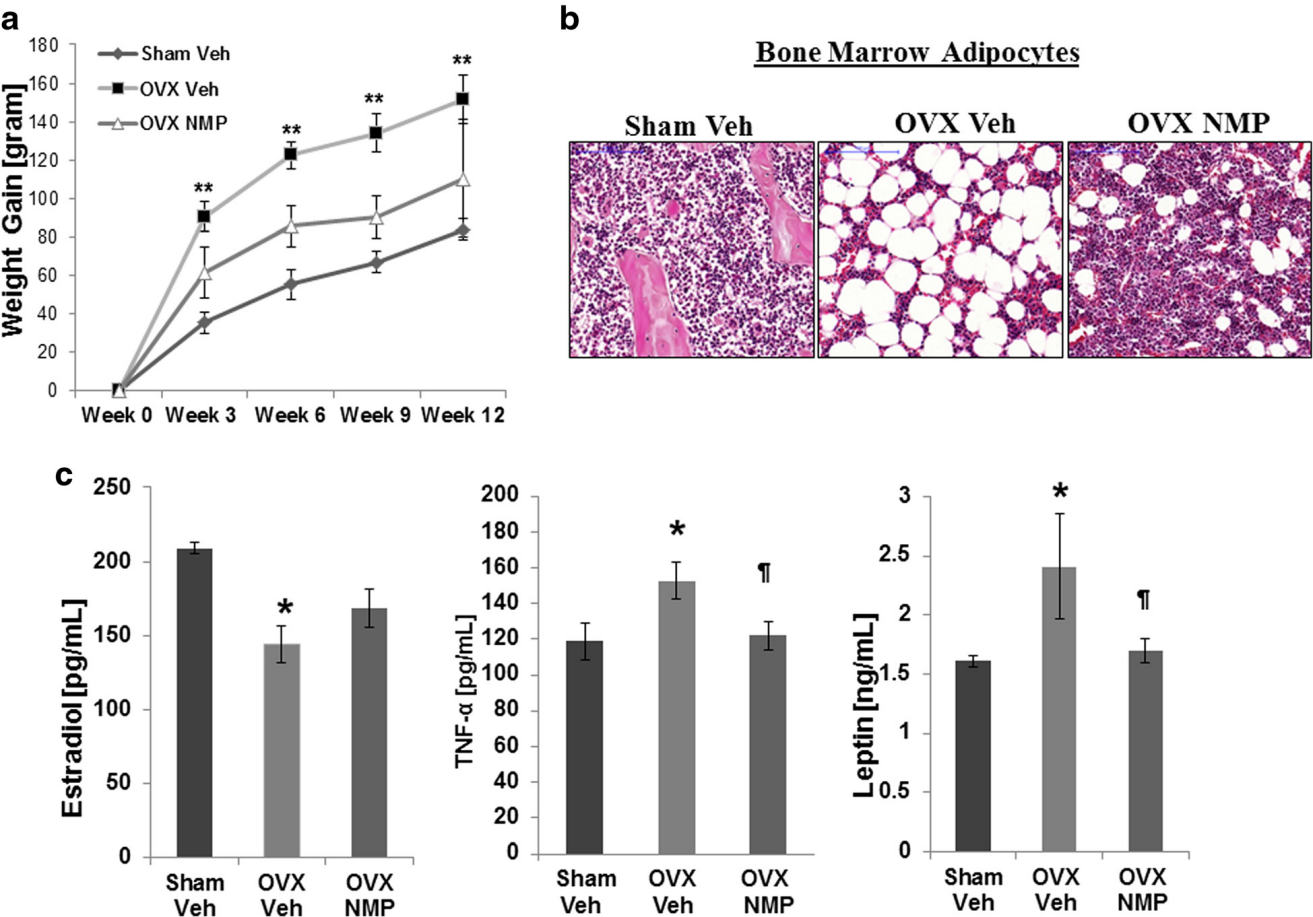
Changes in body weight, marrow adiposity, and biomarkers of the OVX animal model. **a** The animal groups of the study were the following: OVX rats not treated with NMP (OVX Veh), OVX NMP rats treated with 105 μl/100 g/week of NMP, and Sham animals (Sham Veh) not treated with NMP and not ovariectomized. Significant difference in body weight is shown between animal groups from week 3 to week 12 of treatment (Sham Veh vs OVX Veh ** and OVX Veh vs OVX NMP). **b** Representative images of mid-diaphyseal femoral marrow sections from different groups after 15 weeks of treatment. **c** Estradiol, TNF-α, and leptin serum level were also compared between groups (Sham Veh vs OVX Veh * (*P* < 0.05) and OVX Veh vs OVX NMP (*P* < 0.05)). Values are mean ± SD; *error bars* in the figure are presented as SD

Ovariectomy significantly increased the body mass of animals, especially in the first 3 weeks after surgery (first 20 days of the study). OVX animals weighted ~50 g more than their Sham counterparts and only ~30 g more than the OVX NMP group, during these first 3 weeks (Fig. 1a). Over time, the body weight gain increased steadily but was less striking, although a difference of ~70 g was visible between Sham Veh and OVX Veh groups by the end of the study, which equals a 20 % weight increase. While throughout the duration of the treatment, NMP-treated animals gained significantly less weight than the OVX animals (~40 g).

It is known that weight gain after menopause is often due to the decline of estrogen levels. For this reason, we not only measured the body mass of the animals during this treatment but the levels of different biomarkers involved in weight regulation such as estrogen, TNF-α, and leptin were also measured (Fig. 1c). Estradiol level in the OVX Veh group was significantly lower than in Sham Veh group. Even though there was an increase in estradiol level in OVX NMP group, no statistically significant difference was observed between OVX Veh and OVX NMP groups. The serum TNF-α level was significantly more elevated in OVX Veh than in Sham Veh and NMP OVX animals. The same trend was observed for the serum leptin level (Fig. 1c).

The three common findings in osteoporotic bone are increased levels of osteoclastogenesis, decreased osteoblastogenesis, and increased bone marrow adipogenesis. The first is associated with a lack of estrogens and therefore is more commonly seen in women during their postmenopausal years. The reduction in osteoblastogenesis, with the concomitant increase in adipogenesis, is the consequence of the shift in the differentiation of bone marrow cells predominantly into adipocytes [6]. The consequence of these changes is a progressively fatty bone with reduced bone mass. As shown in the Fig. 1b, estrogen deficiency increases fat accumulation in the bone marrow. The NMP treatment of ovariectomized animal counteracts this process.

### Histology of white adipose tissue (WAT) and mRNA PPARγ levels

**Fig. 2:**
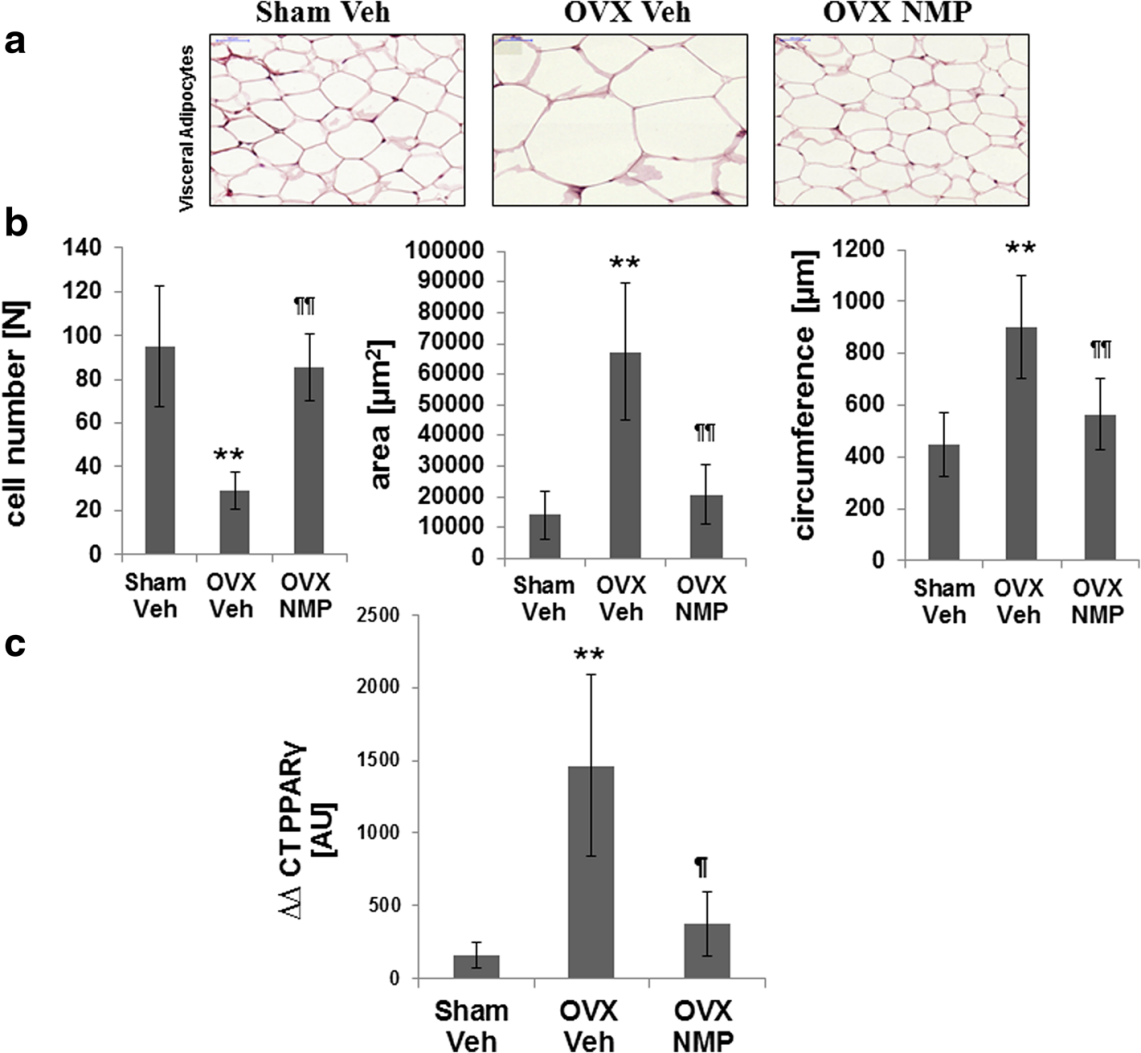
Effects of NMP treatment on histologic sections and PPARγ from WAT of ovariectomized rats. **a** Histology of white adipose tissue (WAT) with hematoxylin and eosin staining. **b** Characteristics of adipocytes in OVX rats analyzed using ImageJ software. **c** The effects of rat ovariectomy and NMP treatment on PPARγ mRNA expression. RT-PCR is presented from samples obtained from Sham-operated (SHAM), ovariectomized (OVX), and ovariectomized rats treated with NMP **P < 0.01 vs. SHAM; *P* < 0.01 vs. OVX

Ovariectomy also resulted in increased size of adipocytes (hypertrophy) from white adipose tissues (Fig. 2a). As seen from the supplementary data, NMP treatment (OVX NMP) led to a reduction of visceral white fat pads. Ovariectomized rats (OVX Veh) showed a significant decrease in the number of adipose cells per area. Similarly, adipocytes from OVX Veh rats had a larger mean cell circumference and area than the adipocytes from the Sham Veh and OVX NMP group (Fig. 2b)

To determine whether the effects of NMP on WAT adipocyte size are associated with changes in PPARγ, we measured mRNA levels of PPARγ in WAT from the animal groups. In the OVX Veh group, PPARγ levels were substantially upregulated. Administration of NMP on the ovariectomized animals significantly decreased PPARγ expression.

### NMP inhibits adipocyte formation from hMSCs

**Fig. 3:**
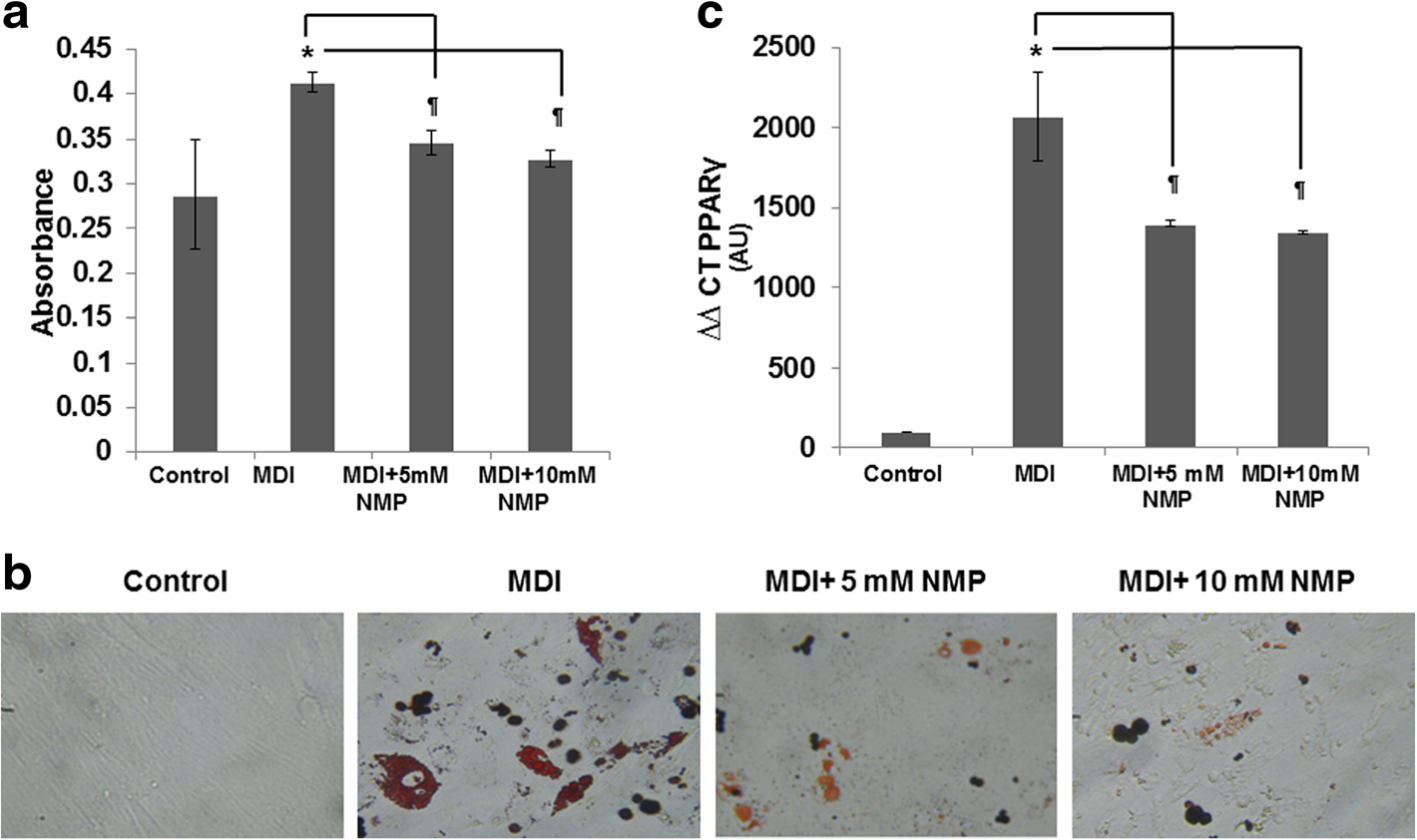
Influence of NMP on adipocyte differentiation from hMCS. **a** Human bone marrow-derived mesenchymal cells (MSCs) were induced into adipogenesis by differentiation medium (MDI) containing IBMX, dexamethasone, indomethacin, and insulin as described in the “Methods” section. After 14 days, the lipid droplets were stained with Oil Red O. For the quantitative analysis, Oil Red O staining was extracted with isopropyl alcohol and the absorbance at ʎ490 nm was measured. Data are presented as mean ± SD. **b** Representative micrographs showing the cell monolayers stained with Oil Red O. As seen from the staining and the quantification in cells treated with differentiation medium alone, there is much more lipid accumulation than in cells treated with both differentiation medium and NMP (5 and 10 mM). However, no significant difference was observed between the two different doses of NMP. **c** Real-time PCR analysis for PPARγ mRNA expression. The levels of PPARγ were normalized to the levels of PPIA, suitable reference genes during adipogenic differentiation of MSCs. Result is presented as ΔΔCT. *P < 0.01 MDI-treated cells vs. control; *P* < 0.05 NMP + MDI-treated cells vs. MDI-treated cells

Mesenchymal stem cells (MSCs) can differentiate into several lineages, including adipocytes [26, 27]. To induce adipogenic differentiation, sub-confluent (70 %) human mesenchymal stem cells (hMSCs) were seeded in 12-well plates, cultured, and treated for 14 days in adipogenic medium (MDI). hMSCs were induced to differentiate into mature adipocytes, and lipid accumulation was assessed by Oil Red O staining (Fig. 3b). Lipid accumulation in MDI-treated group was evident compared to the control group. Cells co-treated with MDI and with 5 mM NMP or 10 mM NMP show less lipid accumulation than the MDI-induced cells. This anti-adipogenic effect of NMP was also observed when the absorbance of the Oil Red O staining was measured (Fig. 3a), displaying a significant difference between MDI-induced and MDI/NMP-treated cells. Consistently, expression level of PPARγ, a crucial adipogenic marker which plays a central role in adipocyte gene expression and differentiation, was also significantly reduced upon NMP treatment (Fig. 3c).

### NMP inhibits adipocyte maturation

**Fig. 4:**
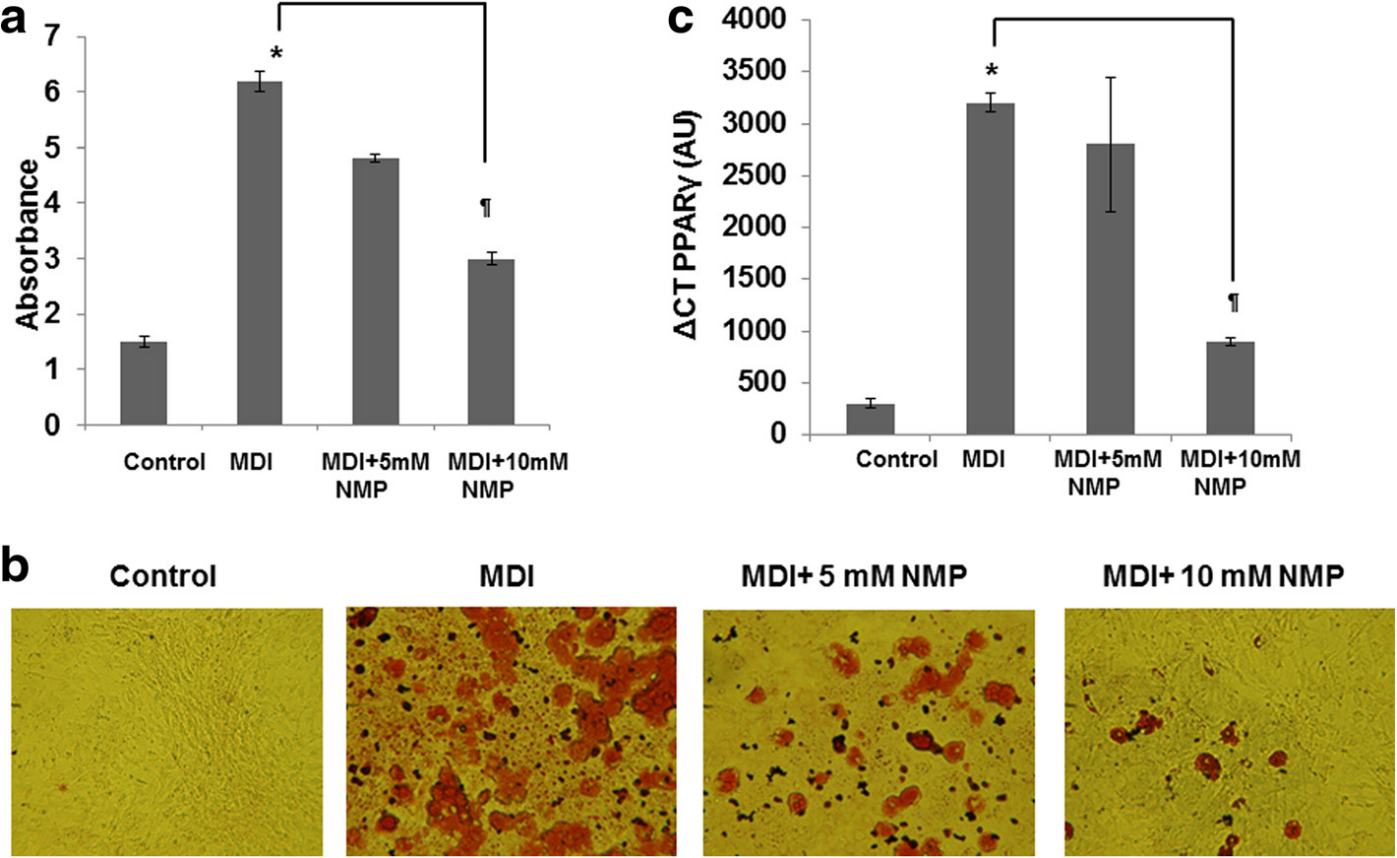
Influence of NMP on T3-L1 adipogenic maturation. **a** Two-day post-confluent 3T3-L1 pre-adipocytes were cultured with differentiation medium (MDI) in the absence or presence of NMP (5 or 10 mM) for 10 days. **b** Representative micrographs showing the cell monolayers stained with Oil Red O. For the quantitative analysis, Oil Red O staining was extracted with isopropyl alcohol and the absorbance at ʎ490 nm was measured. Data are presented as mean ± SD. **c** Real-time PCR was used to quantify the effect of NMP on adipogenic gene expression (PPARγ). On day 8, PPARγ mRNA expression was analyzed by real-time PCR. Result is presented as ΔΔCT. *P < 0.01 MDI-treated cells vs. control; *P* < 0.01 NMP + MDI-treated cells vs. MDI-treated cells

To study the effect of NMP on the differentiation of pre-adipocytes to mature adipocytes, we employed 3T3-L1 cells. These cells are a well-established pre-adipocyte cell line used for the investigation of pre-adipocytes conversion into adipocytes. Post-confluent 3T3-L1 pre-adipocytes were treated with adipogenic medium (MDI) then treated with different doses of NMP (5 and 10 mM) or without NMP for 10 days. To determine the effect of NMP on adipogenic differentiation, the accumulated intracellular lipid was stained with Oil Red O dye and quantified. Oil Red O staining performed on day 10 of the treatment shown in Fig. 4b suggests that 3T3-L1 pre-adipocytes differentiate into mature adipocytes. However, cells co-treated with 5 mM NMP for 10 days showed inhibition of MDI-induced lipid droplet accumulation and 10 mM NMP nearly fully blocked lipid droplet accumulation. As shown in Fig. 4a, a dose-dependent reduction in lipid accumulation was observed in the cells treated with NMP, suggesting that NMP inhibits adipocyte transcription factors during adipocyte differentiation. To further evaluate the effect of NMP on adipocyte formation, the expression level of PPARγ was investigated. The mRNA levels of PPARγ were significantly lower in 10-mM NMP-treated 3T3-L1 pre-adipocytes compared to 5-mM NMP or MDI alone treated cells (Fig. 4c).

### Effect of NMP on the binding ability of BET bromodomains

**Fig. 5:**
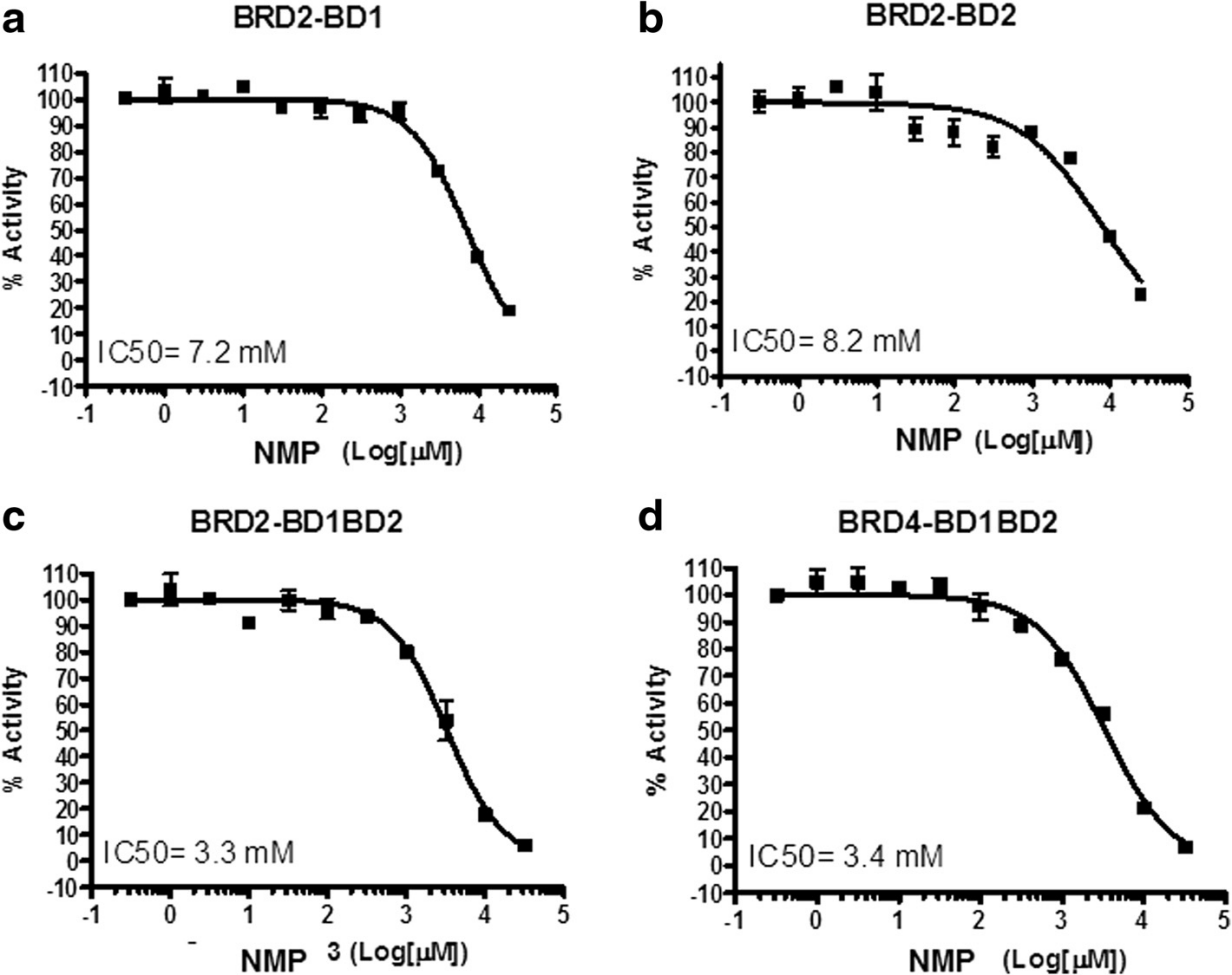
NMP binding to bromodomains. The effect of NMP on the binding of diverse recombinant BET bromodomains using the AlphaScreening assay. The millimolar half-maximum inhibitory concentration (IC50) of NMP is provided for each protein. **a** BRD2 BD1; **b** BRD2 BD2; **c** BRD2 BD1BD2; **d** BRD4 BD1BD2)

To assess the possibility of NMP acting as a bromodomain inhibitor particularly for BRD2 and BRD4, both associated with adipogenesis, we used an AlphaScreen assay format to determine the effect of NMP on recombinant human BET bromodomains. Our results suggest that NMP inhibits the binding of acetyl-lysines to BRD2-BD1BD2, a member of the “BET” subfamily of bromodomain-containing proteins [29]. AlphaScreen dose response experiments performed against BRD2 and BRD4 as a whole and BRD2 BD1 and BRD2 BD2 component independently gave raise to millimolar half-maximum inhibitory concentration (IC50) values for NMP (Fig. 5). Affinity to BRD2 and BRD4 as a whole are 3.3 and 3.4 mM, respectively, and therefore very similar.

## Discussion

In an ovariectomized rat model, mimicking menopause, we recently showed that N-methyl pyrrolidone (NMP) prevents bone loss and improves both mass and quality of bones [24]. In the present study, we examined whether NMP could reduce obesity and obesity-related metabolic disorders in ovariectomized rats. During menopausal transition, many women gain weight and weight gain risks such as high blood pressure, heart diseases, and diabetes. Overweight and obesity in menopausal women are important public health concerns especially in an aging society [13]. In our study, ovariectomy induced a decrease in estradiol level which was accompanied by an increase in marrow fat content. NMP administration was not able to significantly restore estradiol level; however, fat fraction in the bone marrow and abdominal fat was markedly decreased by the treatment compared to ovariectomized non-treated group. This suggests that NMP acts via an estrogen-independent mechanism.

Moreover, the weight gain and abdominal fat accumulation in postmenopausal women cause a higher risk for insulin resistance [30, 31]. Additionally, clinical data have shown that other cytokines, such as TNF-α, leptin, and adiponectin, are also involved in insulin resistance [32, 33]. In our study, NMP treatment reversed the effect of ovariectomy on serum TNF-α and leptin levels. These results are in agreement with the fact that NMP decreases body weight gain and that in humans, TNF-α and leptin are known to increase with body mass index [34].

Another consequence of estrogen deficiency is white adipose tissue (WAT) accumulation [3]. Indeed, in the present study, ovariectomy induced an increase in the area and circumference of adipocytes compared to Sham group. NMP treatment was able to reverse the area and circumference alterations suggesting that NMP reduces the total fat mass probably by inhibiting adipocyte differentiation and maturation. This hypothesis is supported by the fact that osteoblasts and adipocytes differentiate from the same progenitor and that we have already demonstrated that NMP favors osteoblast differentiation and bone regeneration [23, 24]. Different studies showed that in addition to stimulating osteoblast differentiation and bone formation, the suppression of adipogenesis may be of considerable significance to prevent or treat osteoporosis [35, 36]. In support of this notion, it has been shown that alendronate, a drug used to treat osteoporosis in postmenopausal women, can delay adipogenesis and enhance osteogenesis by reducing the expression of PPARγ, the adipogenic transcription factor, in human mesenchymal stem cells [37].

Adipogenesis is initiated by the production of the key transcription factor PPARγ, which is responsible for inducing the expression of adipocyte-specific genes [15]. In this study, mRNA expression level of PPARγ in adipose tissue was tenfold higher in ovariectomized rats compared to Sham group. Consistent with the effects of NMP on ovariectomy-induced fat accumulation and marrow adipogenesis, NMP decreased the expression of PPARγ in WAT. In vitro, NMP strongly inhibited the hMSCs and 3T3-L1 pre-adipocyte differentiation as indicated by reduction in Oil Red O staining and decrease of PPARγ gene expression.

Recently, we showed that NMP acts as a low affinity bromodomain inhibitor for BRD2, BRD3, BRD4, and BRDT and has potential for osteoporosis treatment [24]. BRD2, a member of the BET family shows anti-adipogenic function in a hypomorphic mouse model and in 3T3-L1 pre-adipocytes [38, 39]. BRD2 knock-out causes severe obesity [38]. Therefore, one would expect that BRD2 inhibition would induce weight gain. Our results, however, show that NMP despite its affinity to both bromodomains of BRD2 (Fig. 5) prevents weight gain caused by estrogen deficiency. This is in line with weight loss being the sole side effect of JQ1 application for contraception in male mice [40], where JQ1 was employed as a high affinity bromodomain inhibitor targeting BRD2, BRD3, BRDT, and preferentially BRD4 [41]. Just recently, Jumonji domain-containing protein 6 (JMJD6) was found to be a nuclear protein involved in histone modification, transcription, and RNA processing and crucial during adipogenesis [21]. JMJD6 directs transcriptional activation of PPARγ2 during adipocyte differentiation dependent on BRD4 [21]. Therefore, inhibition of the scaffolding capability of BRD4 by bromodomain inhibitors JQ1 and NMP, as shown here, prevents transcriptional activation of PPARγ2 and in consequence adipogenesis. BRD2 inhibition, in this context, appears of minor importance.

Our results show the novel action of NMP as a modulator of fat accumulation and adipocyte differentiation in vivo and in vitro. Epigenetic therapy with small molecules which act as bromodomain inhibitors such as NMP may be an effective strategy to lower adipogenic activity. This suggests that small molecules like NMP which regulate epigenetic alterations are valuable tools for treatment of various diseases and conditions. Particularly, research efforts should focus on the role of small molecules which due to their bromodomain inhibitor properties can act as pluripotent epigenetic therapies for a wide range of diseases.

## Conclusions

The low affinity bromodomain inhibitor NMP inhibits the adipogenic effect of estrogen deficiency at the level of PPARγ expression by inhibition of BRD4.

## Methods

### Animals and treatment

Female Sprague-Dawley (SD) rats (wt. 230 ± 10 g) were obtained from Charles River laboratories. The animals were adapted to laboratory environment for 2 weeks. In three independent experiments, a total of 30 animals were used. The acclimatized rats underwent either bilateral laparotomy (Sham Veh, *N*_total_ = 10) or bilateral ovariectomy (OVX, *N*_total_ = 20). In the first two experiments, three animals per group Sham rats treated with PBS (Sham_Veh), ovariectomized rats treated with PBS (OVX_veh), and ovariectomized rats treated with NMP (OVX_NMP) were used. In the third experiment, four animals per group were used. In total, we had ten animals per group from three independent experiments.

After recovering from surgery for a week, the OVX rats were divided into two groups: OVX with vehicle (OVX Veh, *N*_total_ = 10) and OVX with NMP (OVX NMP, 1/3 of LD50 = 105 μl/100 g/week, equals an overall concentration of 10.5 mM, *N*_total_ = 10). Treatment consisted of intraperitoneal injection of NMP initiated 1 week after and lasted for 15 weeks. The body mass of each rat was monitored weekly, and the administered dose was adjusted accordingly. All animal procedures were approved by the Animal Ethics Committee of the local authorities (Canton Zurich, 40/2012).

Blood sample was collected via abdominal aorta puncture immediately following sacrifice by CO_2_ asphyxiation. Then, a serum specimen was harvested after centrifugation (2000 rpm for 20 min). Samples were stored at −80 °C until further testing and analysis. White adipose tissues and femurs were also removed, fixed, and embedded in paraffin (Sophistolab AG, Muttenz, Switzerland). All samples were then stained with H&E. Femur samples were analyzed for bone marrow adiposity while white fat tissues were analyzed for different known adipocytes parameters. One part of freshly excised adipose tissue was immediately dissected, cut into small pieces suitable for rapid penetration by the RNA*later* RNA Stabilization Reagent (Qiagen GmbH, Hilden, Germany), and submerged according to the manufacturer’s protocol.

### Serological analysis of different markers

Serum markers were analyzed by ELISA to monitor the NMP treatment effect on different metabolic markers. Serum concentration of estradiol (Takara Bio Europe, France), tumor necrosis factor alpha (TNFα), and leptin (Uscn Life Science, Cologne, Germany) were measured according to the manufacturer’s instructions.

### AlphaScreening assay

AlphaScreening assay was performed using recombinant bromodomains and bromodomain ligands or recombinant BET bromodomains and BET ligands from BPS Bioscience (San Diego, USA). The AlphaScreening signal from the assay is correlated with the amount of bromodomain/BET ligand binding to the bromodomain. AlphaScreening signal was measured using EnSpire Alpha 2390 Multilabel reader (Perkin Elmer). Binding experiments were performed in duplicate. AlphaScreening data were analyzed using the computer software, Graphpad Prism. In the absence of the compound, the AlphaScreening signal (*A*_t_) in each data set was defined as 100 % activity. In the absence of the bromodomain/BET ligand, the AlphaScreening signal (*A*_b_) in each data set was defined as 0 % activity. The percent activity in the presence of each compound was calculated according to the following equation: % activity = [(*A* − *A*_b_)/(*A*_t_ − *A*_b_)] × 100, where *A* = AlphaScreening signal in the presence of the compound, *A*_b_ = AlphaScreening signal in the absence of the bromodomain/BET Ligand, and *A*_t_ = AlphaScreening signal in the absence of the compound. The percent inhibition was calculated according to the following equation: % inhibition = 100 − % activity. Values of % activity versus a series of compound concentrations were then plotted using non-linear regression analysis of Sigmoidal dose–response curve generated with the equation *Y* = *B*+(*T* − *B*)/1 + 10^((LogEC50 − *X*) × Hill Slope)^, where *Y* = percent activity, *B* = minimum percent activity, *T* = maximum percent activity, *X* = logarithm of compound, and Hill Slope = slope factor or Hill coefficient. The IC50 value was determined by the concentration causing a half-maximal percent activity.

### Cell culture and differentiation of hMSCs

Human mesenchymal stromal cells derived from bone marrow were provided by the Ehrbar Laboratory at University Hospital Zurich. The cells were cultured as previously described in [42]. For adipogenic differentiation, cells were plated at 4 × 10^3^ cells/cm^2^ and cultured in α-MEM medium with 10 % fetal bovine serum (FBS), 0.5 mM isobutyl-methylxanthine (IBMX), 1 μM dexamethasone, 10 μM insulin, and 100 μM indomethacin. The medium was changed every 2 days, and after 14 days, fat droplet formation was analyzed by Oil Red staining using microscopy and semiquantitative analysis at λ490 nm using a Synergy HT ELISA microplate reader (BioTek)

### Cell culture and differentiation of 3T3-L1 cells

The 3T3-L1 cells were cultured in Dulbecco’s modified Eagle’s medium (DMEM; Invitrogen, Carlsbad, CA, USA) with 10 % FBS at 37 °C in 5 % CO_2_. The cells were seeded at a density of 4 × 10^5^ cells/well into a six-well plate. At 2 days post-confluence (day 0), the cells were exposed to an MDI solution (induction solution) containing 0.5 mM IBMX, 1 μM of dexamethasone, and 10 μg/ml of insulin for 2 days. Following induction, cells were switched to a solution containing complete DMEM medium supplemented with 1 μg/ml insulin (maintaining solution). The medium was changed every 2 days for a total of 10 days. To examine the effects of NMP on the differentiation of pre-adipocytes into adipocytes, the cells were treated with various concentrations of NMP at 2 days post-confluence (day 0) and each time, the medium was changed. The fat droplet formation was analyzed by Oil Red staining using microscopy and semiquantitative analysis at λ490 nm using a Synergy HT ELISA microplate reader (BioTek).

### Oil Red O staining

For quantification, cells were fixed with 10 % neutral formalin for 1 h at room temperature, washed with phosphate-buffered saline (PBS), and then stained for 1 h with 0.5 % Oil Red O in 60 % isopropanol. After washing with distilled water, the stained cells were observed under a microscope. The stained lipid droplets were then extracted with isopropanol for quantification by measuring its absorbance at 490 nm.

### RT-PCR

Samples of the dissected adipose tissue (100 mg) in weight were prepared and processed with RNeasy Mini Kit and RNeasy Lipid Tissue Kit (both QIAGEN GmbH, Hilden, Germany) according to the manufacturer’s protocols. The mRNA was reverse-transcribed, and resultant cDNA was subjected to real-time PCR with gene-specific primers using iQ SYBR Green Supermix and an iCycler real-time PCR machine (both from Bio-Rad, Cressier, Switzerland) according to the manufacturer’s instructions. All primers were purchased from Qiagen. Relative gene expression was analyzed using 2^−∆∆*C*t^ method as previously described [43].

### Histology

Paraffin sections of rat femurs and white adipose tissues collected for histological analysis were prepared and stained with hematoxylin and eosin (H&E) as formerly described [23]. Five sections from five different animals (chosen randomly) were examined for changes. The sections were visualized with a Leitz Dialux20 microscope and images captured using a Leica camera.

### Statistical analysis

All statistical analyses were performed with IBM SPSS statistics 22. Data from all parameters were normally distributed (Shapiro–Wilk test). Results are expressed as the mean ± SD and were compared by ANOVA and Student’s *t* test. Results were considered significantly different for *P* < 0.05.

## Availability of supporting data

Additional file 1: Effect of NMP visceral adipose tissue: OVX increases visceral fat and this increase can be reversed by NMP treatment.

## Additional file

Additional file 1: Figure S1. Effect of NMP visceral adipose tissue: Remarkable difference is noticed in visceral adipose tissue (VAT) between (**A**) Sham Veh group (control) and (**B**) OVX Veh group during sacrifice. While, in (**C**) OVX NMP group the visceral adipose tissue content is similar to the control group. (TIF 1881 kb)

## Abbreviations

BD: bromodomain
BET: bromodomain and extraterminal protein
BMD: bone mineral density
BMSCs: bone marrow mesenchymal stem cells
BRD2/4: bromodomain and extraterminal domain protein
hMSCs: human mesenchymal stem cells
LPS: lipopolysaccharide
NMP: N-methyl pyrrolidone
OVX: ovariectomized
PPARγ: peroxisome proliferator-activated receptor gamma
TNF-α: tumor necrosis factor alpha
WAT: white adipose tissue

## References

[1] Rachner TD, Khosla S, Hofbauer LC (2011). Osteoporosis: now and the future. Lancet.

[2] van Seumeren I (2000). Weight gain and hormone replacement therapy: are women’s fears justified?. Maturitas.

[3] Clegg DJ (2012). Minireview: the year in review of estrogen regulation of metabolism. Mol Endocrinol.

[4] Rosen CJ, Bouxsein ML (2006). Mechanisms of disease: is osteoporosis the obesity of bone?. Nat Clin Pract Rheum.

[5] James AW (2013). Review of signaling pathways governing MSC osteogenic and adipogenic differentiation. Scientifica (Cairo).

[6] Chen Q, Shou P, Zheng C, Jiang M, Cao G, Yang Q et al. Fate decision of mesenchymal stem cells: adipocytes or osteoblasts[quest]. Cell Death Differ. 2016. doi:10.1038/cdd.2015.168 10.1038/cdd.2015.168 PMC4946886 26868907

[7] Li GW, Xu Z, Chen QW, Chang SX, Tian YN, Fan JZ (2013). The temporal characterization of marrow lipids and adipocytes in a rabbit model of glucocorticoid-induced osteoporosis. Skeletal Radiol.

[8] Patsch JM, Li X, Baum T, Yap SP, Karampinos DC, Schwartz AV (2013). Bone marrow fat composition as a novel imaging biomarker in postmenopausal women with prevalent fragility fractures. J Bone Miner Res.

[9] Kanaley JA, Sames C, Swisher L, Swick AG, Ploutz-Snyder LL, Steppan CM (2001). Abdominal fat distribution in pre- and postmenopausal women: the impact of physical activity, age, and menopausal status. Metabolism.

[10] Douchi T, Yamamoto S, Yoshimitsu N, Andoh T, Matsuo T, Nagata Y (2002). Relative contribution of aging and menopause to changes in lean and fat mass in segmental regions. Maturitas.

[11] Mauvais-Jarvis F (2011). Estrogen and androgen receptors: regulators of fuel homeostasis and emerging targets for diabetes and obesity. Trends Endocrinol Metab.

[12] Poledne R, Lorenzova A, Stavek P, Valenta Z, Hubacek J, Suchanek P (2009). Proinflammatory status, genetics and atherosclerosis. Physiol Res.

[13] Lizcano F, Guzman G (2014). Estrogen deficiency and the origin of obesity during menopause. Biomed Res Int.

[14] Krum SA, Brown M (2008). Unraveling estrogen action in osteoporosis. Cell Cycle.

[15] Rosen ED, Hsu CH, Wang X, Sakai S, Freeman MW, Gonzalez FJ (2002). C/EBPalpha induces adipogenesis through PPARgamma: a unified pathway. Genes Dev.

[16] Jaenisch R, Bird A (2003). Epigenetic regulation of gene expression: how the genome integrates intrinsic and environmental signals. Nat Genet.

[17] Marchand JR, Caflisch A (2015). Binding mode of acetylated histones to bromodomains: variations on a common motif. ChemMedChem.

[18] Belkina AC, Denis GV (2012). BET domain co-regulators in obesity, inflammation and cancer. Nat Rev Cancer.

[19] Belkina AC, Nikolajczyk BS, Denis GV (2013). BET protein function is required for inflammation: Brd2 genetic disruption and BET inhibitor JQ1 impair mouse macrophage inflammatory responses. J Immunol.

[20] Huang H, Zhang J, Shen W, Wang X, Wu J, Wu J (2007). Solution structure of the second bromodomain of Brd2 and its specific interaction with acetylated histone tails. BMC Struct Biol.

[21] Hu YJ, Belaghzal H, Hsiao WY, Qi J, Bradner JE, Guertin DA (2015). Transcriptional and post-transcriptional control of adipocyte differentiation by Jumonji domain-containing protein 6. Nucleic Acids Res.

[22] Ghayor C, Correro RM, Lange K, Karfeld-Sulzer LS, Gratz KW, Weber FE (2011). Inhibition of osteoclast differentiation and bone resorption by N-methylpyrrolidone. J Biol Chem.

[23] Miguel BS, Ghayor C, Ehrbar M, Jung RE, Zwahlen RA, Hortschansky P (2009). N-methyl pyrrolidone as a potent bone morphogenetic protein enhancer for bone tissue regeneration. Tissue Eng Part A.

[24] Gjoksi B, Ghayor C, Siegenthaler B, Ruangsawasdi N, Zenobi-Wong M, Weber FE (2015). The epigenetically active small chemical N-methyl pyrrolidone (NMP) prevents estrogen depletion induced osteoporosis. Bone.

[25] Ghayor C, Gjoksi B, Siegenthaler B, Weber FE (2015). N-methyl pyrrolidone (NMP) inhibits lipopolysaccharide-induced inflammation by suppressing NF-kappaB signaling. Inflamm Res.

[26] Caplan AI (1991). Mesenchymal stem cells. J Orthop Res.

[27] Pittenger MF, Mackay AM, Beck SC, Jaiswal RK, Douglas R, Mosca JD (1999). Multilineage potential of adult human mesenchymal stem cells. Science.

[28] Astudillo P, Rios S, Pastenes L, Pino AM, Rodriguez JP (2008). Increased adipogenesis of osteoporotic human-mesenchymal stem cells (MSCs) characterizes by impaired leptin action. J Cell Biochem.

[29] Florence B, Faller DV (2001). You bet-cha: a novel family of transcriptional regulators. Front Biosci.

[30] Folsom AR, Kushi LH, Anderson KE, Mink PJ, Olson JE, Hong CP (2000). Associations of general and abdominal obesity with multiple health outcomes in older women: the Iowa Women’s Health Study. Arch Intern Med.

[31] Toth MJ, Tchernof A, Sites CK, Poehlman ET (2000). Menopause-related changes in body fat distribution. Ann N Y Acad Sci.

[32] de Lartigue G, Barbier de la Serre C, Espero E, Lee J, Raybould HE (2011). Diet-induced obesity leads to the development of leptin resistance in vagal afferent neurons. Am J Physiol Endocrinol Metab.

[33] Stefan N, Vozarova B, Funahashi T, Matsuzawa Y, Weyer C, Lindsay RS (2002). Plasma adiponectin concentration is associated with skeletal muscle insulin receptor tyrosine phosphorylation, and low plasma concentration precedes a decrease in whole-body insulin sensitivity in humans. Diabetes.

[34] Takahashi M, Funahashi T, Shimomura I, Miyaoka K, Matsuzawa Y (1996). Plasma leptin levels and body fat distribution. Horm Metab Res.

[35] Gimble JM, Zvonic S, Floyd ZE, Kassem M, Nuttall ME (2006). Playing with bone and fat. J Cell Biochem.

[36] Zhang JF, Fu WM, He ML, Wang H, Wang WM, Yu SC (2011). MiR-637 maintains the balance between adipocytes and osteoblasts by directly targeting Osterix. Mol Biol Cell.

[37] Duque G, Rivas D (2007). Alendronate has an anabolic effect on bone through the differentiation of mesenchymal stem cells. J Bone Miner Res.

[38] Wang F, Liu H, Blanton WP, Belkina A, Lebrasseur NK, Denis GV (2010). Brd2 disruption in mice causes severe obesity without type 2 diabetes. Biochem J.

[39] Zang K, Wang J, Dong M, Sun R, Wang Y, Huang Y (2013). Brd2 inhibits adipogenesis via the ERK1/2 signaling pathway in 3T3-L1 adipocytes. PLoS One.

[40] Matzuk MM, McKeown MR, Filippakopoulos P, Li Q, Ma L, Agno JE (2012). Small-molecule inhibition of BRDT for male contraception. Cell.

[41] Filippakopoulos P, Qi J, Picaud S, Shen Y, Smith WB, Fedorov O (2010). Selective inhibition of BET bromodomains. Nature.

[42] Qian S-W, Li X, Zhang Y-Y, Huang H-Y, Liu Y, Sun X (2010). Characterization of adipocyte differentiation from human mesenchymal stem cells in bone marrow. BMC Dev Biol.

[43] Livak KJ, Schmittgen TD (2001). Analysis of relative gene expression data using real-time quantitative PCR and the 2(-Delta Delta C(T)) method. Methods.

