# Supplementary figures and images for: The bromodomain inhibitor N-methyl pyrrolidone reduced fat accumulation in an ovariectomized rat model
Source: Clin Epigenetics. 2016 Apr 22;8:42. doi: 10.1186/s13148-016-0209-2 (PMC4840488; doi:10.1186/s13148-016-0209-2)

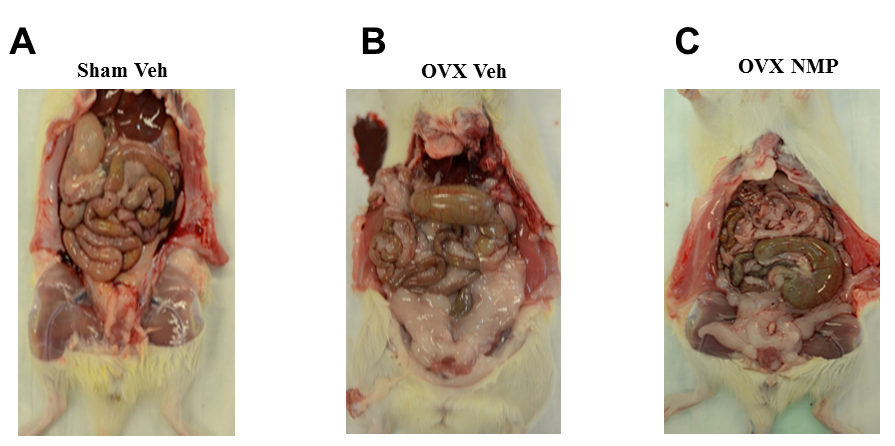

Supplement: Additional file 1: Figure S1. — Effect of NMP visceral adipose tissue: Remarkable difference is noticed in visceral adipose tissue (VAT) between (A) Sham Veh group (control) and (B) OVX Veh group during sacrifice. While, in (C) OVX NMP group the visceral adipose tissue content is similar to the control group. (TIF 1881 kb) [file 13148_2016_209_MOESM1_ESM.tif]
